# Selective somatostatin receptor 5 inhibition improves hepatic insulin sensitivity

**DOI:** 10.1002/prp2.1043

**Published:** 2022-12-30

**Authors:** Yumiko Okano Tamura, Jun Sugama, Shin‐ichi Abe, Yuji Shimizu, Hideki Hirose, Masanori Watanabe

**Affiliations:** ^1^ Cardiovascular and Metabolic Drug Discovery Unit Takeda Pharmaceutical Company Limited Fujisawa Japan; ^2^ Biomolecular Research Laboratories Takeda Pharmaceutical Company Limited Fujisawa Japan

**Keywords:** hepatic insulin resistance, somatostatin, SSTR5, type 2 diabetes

## Abstract

Diabetes is a metabolic disorder with an increasing global prevalence. Somatostatin (SST), a peptide hormone, regulates hormone secretion via five SST receptor (SSTR) subtypes (SSTR1–5) in a tissue‐specific manner. As SSTR5 is expressed in pancreatic β‐cells and intestinal L‐cells, studies have suggested that SSTR5 regulates glucose tolerance through insulin and incretin secretion, thereby having a prominent role in diabetes. Moreover, SSTR5 knockout (KO) mice display enhanced insulin sensitivity; however, the underlying mechanism has not been clarified. Therefore, in this study, we investigate the effect of SSTR5 blockade on insulin resistance and the target organ using SSTR5 KO mice and a selective SSTR5 antagonist (compound‐1). High‐fat diet (HFD)‐fed SSTR5 KO mice exhibited significantly lower homeostasis model assessment of insulin resistance (HOMA‐IR) than HFD‐fed wild‐type mice. Two‐week oral administration of compound‐1 dose‐dependently and significantly reduced changes in the levels of glycosylated hemoglobin (GHb), plasma glucose, plasma insulin, and HOMA‐IR in male KK‐A^y^/Ta Jcl mice (KK‐A^y^ mice), a model of obese type 2 diabetes with severe insulin resistance. Additionally, compound‐1 significantly increased the glucose infusion rate while decreasing hepatic glucose production in male KK‐A^y^ mice, as evidenced by hyperinsulinemic‐euglycemic clamp analyses. In addition, compound‐1 ameliorated the insulin‐induced Akt phosphorylation suppression by octreotide in the liver of male C57BL/6J mice. Collectively, our results demonstrate that selective SSTR5 inhibition can improve insulin sensitivity by enhancing liver insulin action; thus, selective SSTR5 antagonists represent potentially novel therapeutic agents for type 2 diabetes.

## Graphical abstract

We demonstrated that i) deletion of SSTR5 in mice suppresses insulin resistance induced by high‐fat diet feeding, ii) an SSTR5 antagonist exhibits antidiabetic effects and improves hepatic insulin sensitivity in obese diabetic KK‐A^y^ mice, and iii) an SSTR5 antagonist alleviates the suppression effect of somatostatin agonist on insulin‐induced Akt phosphorylation in the liver in mice. In conclusion, selective SSTR5 inhibition can improve insulin sensitivity by enhancing liver insulin action.

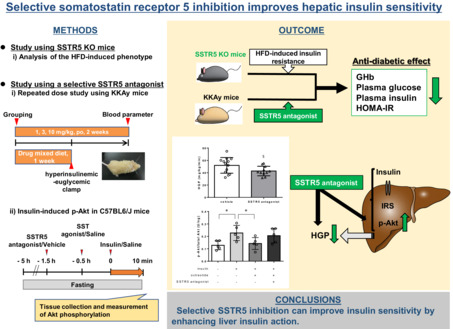

**Abbreviations**
BG: blood glucose
BW: body weight
DPP4: dipeptidyl peptidase 4
FI: food intake
GH: growth hormone
GHb: glycosylated hemoglobin
GIR: glucose infusion rate
GLP‐1: glucagon‐like peptide‐1
HFD: High‐fat diet
HGP: hepatic glucose production
HOMA‐IR: homeostasis model assessment of insulin resistance
HTRF: homogeneous time‐resolved fluorescence
KO: knockout
MC: methyl cellulose
PG: plasma glucose
PI3K: phosphatidyl inositol 3 kinase
PTP: phosphotyrosine phosphatases
Rd: glucose disappearance rate
SST: somatostatin
SSTR: SST receptor
TG: triglyceride
WT: wild‐type

## 1 INTRODUCTION

The global prevalence of type 2 diabetes is steadily increasing. In 2021, the number of patients with diagnosed and undiagnosed type 2 diabetes among adults aged >20 years was approximately 537 million worldwide. By the year 2045, this number is estimated to reach 783 million, i.e., 1 in 10 adults will develop diabetes. Furthermore, according to the 10th edition of the International Diabetes Federation, in 2021, 541 million individuals had developed impaired glucose tolerance, a prediabetic condition; this number is projected to rise to 730 million by 2045.^1^ Thus, type 2 diabetes remains a major global health issue.

Diabetes is a metabolic disease characterized by hyperglycemia due to insufficient insulin secretion, insulin inaction, or both. In diabetes, chronic hyperglycemia is associated with long‐term damage and dysfunction of various organs, resulting in microvascular (e.g., nephropathy, retinopathy, and neuropathy) and macrovascular diseases (e.g., myocardial infarction, stroke, and peripheral arterial disease) associated with a high mortality rate.^2^, ^3^, ^4^

The number of deaths attributable to diabetes was estimated to be 4.2 million in 2019, contributing to 11.3% of deaths worldwide.^5^ Conversely, in a recent large clinical study, appropriate glycemic control by antidiabetic drugs significantly decreased the risk of developing micro‐ and macrovascular diseases, in particular cardiovascular events.^6^, ^7^, ^8^, ^9^

Insulin resistance is an initial indicator of abnormal glucose homeostasis^10^, ^11^ and is a strong risk factor for diabetic complications^12^; thus, insulin sensitizers, including metformin, are used as the first‐line treatment for type 2 diabetes.^13^, ^14^ Notably, a Diabetes Prevention Program study reported that metformin could effectively delay the onset of diabetes^15^ and suppresses diabetes progression by preventing β‐cell exhaustion and death caused by excessive insulin secretion.

Somatostatin (SST), a peptide hormone, was discovered in the hypothalamus as an inhibitor of growth hormone (GH) secretion^16^ and has two biologically active forms, namely a 14 or 28 amino acid peptide hormone (SST14 and SST28, respectively) generated from preproSST (116 amino acids) in mammals.^16^, ^17^, ^18^ SST14 is predominantly expressed in the pancreatic δ cells, stomach, and neural tissues, whereas SST28 is predominantly expressed in the mucosa of the ileum and colon. Both SST14 and SST28 bind to the SST receptor (SSTR), which belongs to the G‐protein‐coupled receptor family containing seven transmembrane domains. To date, five SSTR genes have been cloned (*SSTR1–5*) in humans and other species^19^; *SSTR2* is alternatively spliced to generate two isoforms, SSTR2A and SSTR2B.^20^ These five SSTRs are widely distributed in various tissues, including the brain, pituitary, stomach, liver, pancreas, and intestine.^21^, ^22^ Therefore, SSTs show multiple physiological actions through SSTRs in a tissue‐specific manner, such as the inhibition of GH and thyroid‐stimulating hormone secretion from the anterior pituitary, inhibition of pancreatic endocrine and exocrine secretions, and inhibition and modulation of gastrointestinal functions.^23^

SSTR5 is expressed in the pancreatic β‐cells that secrete insulin^24^, ^25^ and intestinal L‐cells that secrete gut hormones including glucagon‐like peptide‐1 (GLP‐1) and peptide YY.^26^ SST28 binds to SSTR5 with a 10‐fold affinity higher than SST14^27^ and is secreted after meals in humans^28^; consequently, SSTR5 negatively regulates postprandial glucose excursion by inhibiting the secretion of these hormones. Therefore, several SSTR5 antagonists have been developed in the recent decade, and their therapeutic effects on glucose intolerance have been elucidated in animal models.^29^, ^30^, ^31^, ^32^, ^33^, ^34^ Interestingly, SSTR5 deletion has been reported to increase insulin sensitivity; however, the associated mechanisms have not been fully characterized.^35^

In this study, we investigate the effects of SSTR5 inhibition on insulin resistance in vivo. First, we analyze the phenotype of high‐fat diet (HFD)‐fed SSTR5 knockout (KO) mice and evaluate the therapeutic potential of SSTR5 antagonism for diabetic phenotypes, including insulin resistance, using a novel selective SSTR5 antagonist (compound‐1)^36^ in male KK‐A^y^/Ta Jcl mice (KK‐A^y^ mice), a model of obese type 2 diabetes with severe insulin resistance. Finally, we investigate the target organ by which selective SSTR5 antagonists alleviate insulin resistance using a hyperinsulinemic‐euglycemic clamp technique in male KK‐A^y^ mice and measuring insulin‐induced Akt phosphorylation in male C57BL/6J mice. The findings of this study provide deeper insights into the mechanism by which SSTR5 inhibition affects type 2 diabetes and will help to facilitate the development of effective therapeutic strategies against insulin resistance in type 2 diabetes.

## 2 MATERIALS AND METHODS

### 2.1 Materials

**FIGURE 1:**
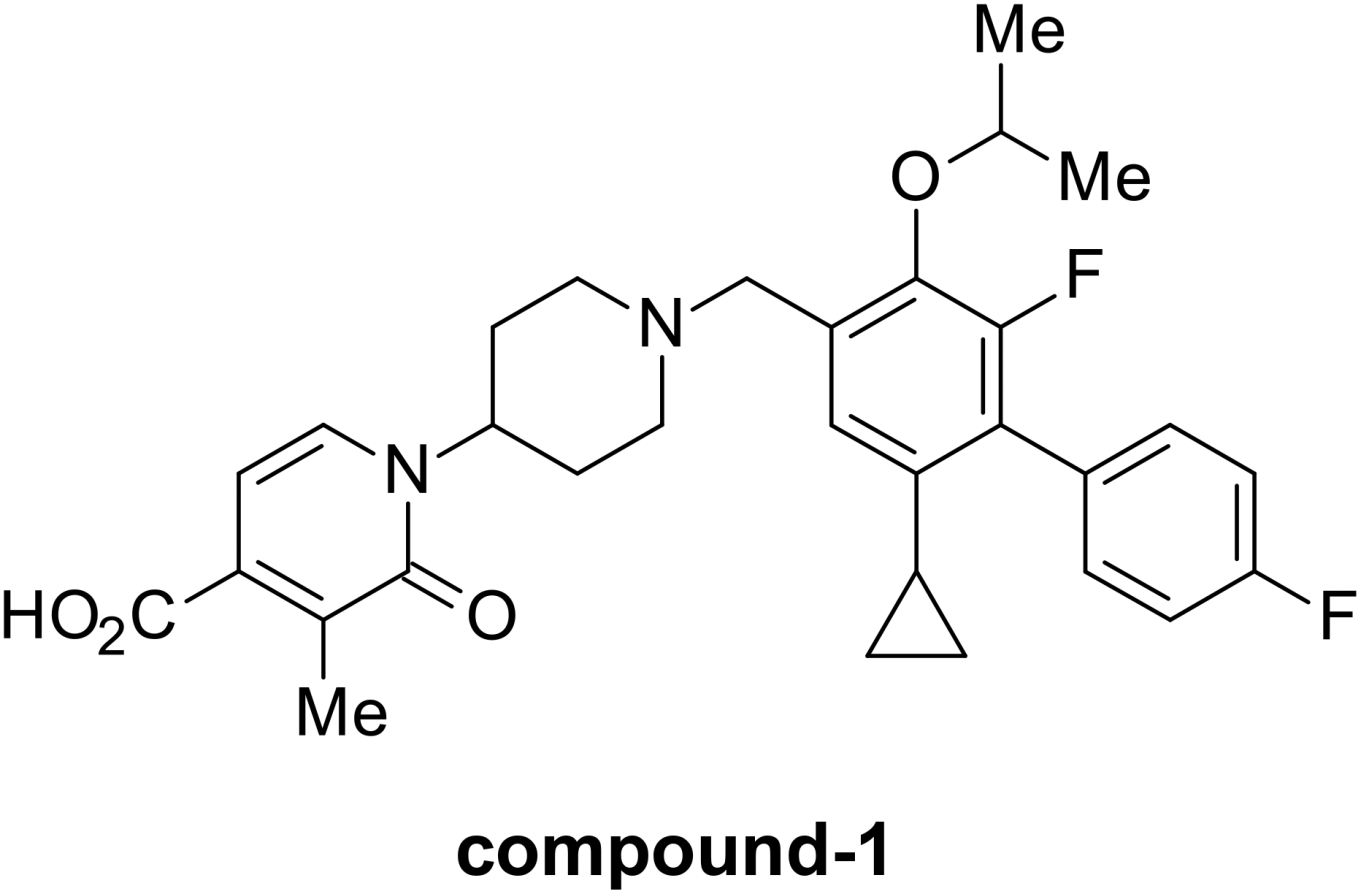
Chemical structure of compound‐1.

Compound‐1 and its hydrochloride salt were synthesized by Takeda Pharmaceutical Company Limited. The chemical structure of compound‐1 is shown in Figure 1. The drugs were either suspended in 0.5% methyl cellulose (MC) solution (FUJIFILM Wako Pure Chemical Corporation) or mixed with CE‐2 powder containing 0.1% cornstarch (Oriental Yeast Co., Ltd.).

### 2.2 SSTR5 cAMP assay

Chinese hamster ovary cells stably expressing human or mouse SSTR5 were suspended in assay buffer Hank's balanced salt solution (Life Technologies) supplemented with 5 mmol/L HEPES (pH 7.5, Life Technologies), 0.1% fatty acid‐free bovine serum albumin (FUJIFILM Wako Pure Chemical Corporation), and 500 μmol/L IBMX (FUJIFILM Wako Pure Chemical Corporation). The cells (4000 cells/well) were incubated with various concentrations of compound‐1 for 15 min at room temperature. Cells were then stimulated with 0.1 nmol/L SST28 (304–91 321; FUJIFILM Wako Pure Chemical Corporation) and 0.3 μmol/L forskolin (063–02193; FUJIFILM Wako Pure Chemical Corporation) for 30 min at room temperature. Intracellular cAMP levels were determined using the homogeneous time resolved‐fluorescence (HTRF) cAMP dynamic 2 kit (Cisbio Bioassays) according to the manufacturer's instructions. The HTRF signal was measured using Envision (PerkinElmer, Inc.). pIC_50_ values and 95% confidence intervals were determined using Prism7 (GraphPad) from the data expressed as a percent of inhibition.

### 2.3 Selectivity analysis of the SSTR subtypes

The activity of compound‐1 on human SSTR subtypes other than SSTR5 was evaluated at a concentration of 10 μmol/L using a radioligand binding assay (Eurofins Panlabs Taiwan, Ltd.).

### 2.4 Animals

The animal experiments were approved by the Institutional Animal Care and Use Committee of Shonan Research Center, Takeda Pharmaceutical Company Limited, in a facility accredited by the American Association for Accreditation of Laboratory Animal Care (AAALAC). Male KK‐A^y^/Ta Jcl mice (KK‐A^y^ mice) and male C57BL/6J mice were purchased from CLEA Japan, Inc. and fed a standard rodent diet (CE‐2; CLEA Japan, Inc.). Male SSTR5 KO mice were purchased from Taconic Biosciences Inc., which were generated by the natural mating of each hetero mouse and backcrossed to C57BL/6J Jcl mice more than thrice by Takeda Pharmaceutical Company Limited. The mice were fed either a CE‐2 diet or HFD (D12492, 60 kcal% fat; Research Diets, Inc.). All animals were housed individually under controlled conditions, including a 12/12 h light/dark cycle at 20–26°C and humidity of 40%–70%; food and tap water were available ad libitum.

### 2.5 HFD‐fed SSTR5 KO study

To evaluate the metabolic parameters under the HFD feeding condition, male SSTR5 KO and wild‐type (WT) littermate mice were fed a CE‐2 diet or a HFD for 9–10 weeks from 8 weeks of age (CE‐2; *n* = 8/group, HFD; *n* = 22/group). At 17–18 weeks of age, body weight (BW) and food intake (FI) were measured, and blood samples were collected from the superficial temporal vein for the measurement of plasma parameters. Regarding FI data, one sample from HFD‐fed WT mice and 2 samples from HFD‐fed SSTR5 KO mice were lost because of a technical error. For the evaluation of insulin sensitivity, SSTR5 KO and WT mice were fed an HFD for 4 weeks from 14 weeks of age (*n* = 40/group). At 18 weeks, blood samples were collected from the tail vein after 6 h of fasting for plasma glucose (PG) and plasma insulin measurement.

### 2.6 Repeated dose study of compound‐1 in KK‐A^y^ mice

Male KK‐A^y^ mice aged 7 weeks were used for the dosage studies. Animals were divided into four groups (*n* = 8/group) based on glycosylated hemoglobin (GHb), PG, insulin, and triglyceride (TG) levels; BW; FI; and change in BW during habituation. Mice were orally administered vehicle (0.5% MC) or compound‐1 at a 1, 3, and 10 mg/kg dosage once daily for 2 weeks (days 0–13). BW and FI were monitored every 1–3 days. On day 14, blood samples were collected from the tail vein of mice under fed or 6 h fasting conditions for the measurement of plasma parameters. Finally, the lean and fat mass were measured using the EchoMRI system (Aloka).

### 2.7 Hyperinsulinemic‐euglycemic clamp study in male KK‐A^y^ mice

At 7 weeks of age, male KK‐A^y^ mice were divided into two groups (vehicle group, *n* = 13; compound‐1 group, *n* = 11) based on their GHb and blood glucose (BG) levels and BW. Mice were fed either a CE‐2 powder diet (vehicle group) or a CE‐2 powder diet containing 0.03% hydrochloride salt of compound‐1. After 1 week of administration, catheters filled with saline containing heparin were inserted into the right common carotid artery and left jugular vein under isoflurane anesthesia the day before the clamp study. The other end of the catheter was exteriorized at the back of the neck. After overnight fasting, a hyperinsulinemic‐euglycemic clamp was performed under conscious and unrestrained conditions. Two of the three catheter lumens were used for infusion of 50% glucose solution and saline containing both insulin and [3‐^3^H]‐glucose (PerkinElmer, Inc.). Insulin and [3‐^3^H]‐glucose were infused for priming (300 mU/kg/min and 25 μCi/kg/min, respectively) (t = 0), followed by continuous infusion at 30 mU/kg/min and 2.5 μCi/kg/min throughout the clamp study. BG level was maintained at ~150 mg/dl by measuring BG via the tail vein with a portable glucose analyzer (Accu‐Chek, Roche Diagnostics Corp.) every 5 min, and glucose infusion was performed if necessary. To determine the specific activity of plasma [3‐^3^H]‐glucose, blood samples were collected from the right common carotid artery at t = 100, 115, and 135 min. The plasma was then deproteinized with Ba(OH)_2_ and ZnSO_4_, dried to remove ^3^H_2_O, resuspended in saline, and counted in a liquid scintillation counter (LSC‐7400, Hitachi Aloka Medical). The glucose infusion rate (GIR), glucose disappearance rate (Rd), and hepatic glucose production (HGP) were calculated using a modified formula based on a previous study.^37^ GIR was calculated by averaging the GIR values during t = 95–135 min. Rd was calculated by averaging the value, which was determined as the ratio of the [3‐^3^H]‐GIR to the specific activity of PG at 100, 115, and 135 min. HGP was calculated by averaging the values, which were determined by subtracting the GIR from Rd at 100, 115, and 135 min.

### 2.8 Insulin‐induced Akt phosphorylation study in male C57BL/6J mice

At 10 weeks of age, male C57BL/6J mice were divided into four groups (*n* = 5/group). Following fasting for 5 h, mice were administered insulin (0.3 U/4 ml/kg) intraperitoneally and sacrificed after 10 min of insulin treatment under isoflurane anesthesia. Compound‐1 (10 mg/10 ml/kg) was administered orally for 1.5 h prior to insulin treatment. Octreotide (100 μg/4 ml/kg) (Sandostatin as octreotide acetate; Novartis Pharmaceuticals, Basel, Switzerland) was administered subcutaneously 30 min prior to insulin treatment. Tissue samples were collected, immediately frozen in liquid nitrogen, and stored at −80 °C for Akt phosphorylation analysis. To extract protein from the tissue sample, 1 ml of the Cell Extraction Buffer (FNN0011; Thermo Fisher Scientific) containing protease inhibitor (P8340; Sigma‐Aldrich), PhosSTOP (04–906‐837; Roche), and PMSF (final concentration of 1 mM) was added to 100 mg tissue and homogenized (27 Hz, 2 min) with zirconia beads. The pS473 Akt and total Akt levels were measured using a commercially available kit (KHO0111 or KHO0101; Thermo Fisher Scientific) according to the manufacturer's instructions. One epididymal fat sample of “insulin + octreotide + compound−1”‐treated mice was lost because of an operational error.

### 2.9 Measurement of plasma parameters

Blood samples were collected from the tail vein or superficial temporal vein under fed or 6 h fasting conditions and centrifuged at 13000 × *g* for 5 min at 4°C to obtain the plasma. PG, TG, plasma total cholesterol, and alanine aminotransferase levels were enzymatically measured using the Autoanalyzer 7180 (Hitachi High‐Technologies Corporation). Fasting PG was enzymatically measured using the Glucose CII Test Wako (FUJIFILM Wako Pure Chemical Corporation). Plasma insulin levels were measured using a sandwich enzyme‐linked immunosorbent assay (Shibayagi, Gunma, Japan, or Morinaga, Kanagawa, Japan). GHb was measured using an automated high‐performance liquid chromatography‐based GHb analyzer (HLC‐723 G8, TOSOH Corporation). The homeostasis model assessment of insulin resistance (HOMA‐IR) was calculated using the following formula^38^:

$$\text{fasting insulin}\;\left(\mathrm{\mu U}/\mathrm{ml}\right)\times \text{fasting glucose}\;\left(\mathrm{mg}/\mathrm{dl}\right)/405$$

Further, if the fasting insulin level was lower than the detection limit (20.28 μU/ml), as seen in three and five mice in the WT and KO groups, respectively (WT; *n* = 3/40, KO; *n* = 5/40), HOMA‐IR was calculated using the following formula:

$$\text{fasting insulin}\;\left(20.28\;\mathrm{\mu U}/\mathrm{ml}\right)\times \text{fasting glucose}\;\left(\mathrm{mg}/\mathrm{dl}\right)/405$$

### 2.10 Statistical analysis

All data represent the mean ± SD. To evaluate the effects of SSTR5 deletion in vivo, statistical differences between HFD‐fed WT mice and HFD‐fed SSTR5 KO mice were assessed using the Student's *t*‐test and Aspin–Welch test. Conversely, to evaluate the effects of compound‐1 in KK‐A^y^ mice, the statistical differences between the vehicle and drug treatments were assessed using the Student's *t*‐test, Aspin–Welch test, and two‐tailed Williams' test. In the insulin‐induced Akt phosphorylation study, Student's *t*‐test or Aspin–Welch test was used to assess the comparisons between the control and insulin groups, insulin and insulin + octreotide groups, and between the insulin + octreotide and insulin + octreotide + compound‐1 groups. A closed procedure was used to maintain the overall type‐I error rate at 0.05. That is, the comparison between the control and insulin groups was assessed. If the resulting two‐sided *p*‐value was <0.05, the comparison between the insulin and insulin + octreotide groups was assessed. Next, if the two‐sided *p*‐value was <.05, the comparison between the insulin + octreotide and insulin + octreotide + compound‐1 groups was assessed. For all tests, *p* < .05 was considered statistically significant. Statistical analysis was performed using SAS systems version 8.2 (SAS Institute Inc.) or EXSUS version 8.0 (CAC Croit Corporation).

### 2.11 Nomenclature of targets and ligands

Key protein targets and ligands in this article are hyperlinked to corresponding entries. in http://www.guidetopharmacology.org, the common portal for data from the IUPHAR/BPS Guide to PHARMACOLOGY,^39^ and are permanently archived in the Concise Guide to PHARMACOLOGY 2019/20.^40^

## 3 RESULTS

### 3.1 Deletion of SSTR5 suppresses insulin resistance induced by HFD feeding

We investigated the effects of SSTR5 deletion on metabolic parameters under HFD feeding conditions in mice. Consistent with a previous report,^35^ no significant difference was observed in HFD FI between WT and SSTR5 KO mice(WT = 2.96 g/day, KO = 2.82 g/day). Although a HFD induced obesity and abnormal glucose metabolism, HFD‐fed SSTR5 KO mice showed significantly decreased PG, plasma insulin, and GHb levels compared with HFD‐fed WT mice (Table 1).

**TABLE 1:** Effects of SSTR5 deletion on BW and plasma parameters under HFD feeding conditions.

Parameter (unit)	CE‐2‐fed WT	CE‐2‐fed SSTR5 KO	HFD‐fed WT	HFD‐fed SSTR5 KO
BW (g)	34.9 ± 3.1	32.0 ± 2.4	45.9 ± 4.6	43.4 ± 5.9
PG (mg/dl)	164.6 ± 15.3	166.6 ± 16.4	228.3 ± 70.6	190.2 ± 30.1^$^
Insulin (ng/ml)	5.6 ± 4.6	4.6 ± 2.9	87.5 ± 95.3	32.5 ± 47.8^$^
GHb (%)	4.3 ± 0.3	4.0 ± 0.2*	4.8 ± 0.7	4.0 ± 0.4^$$$^
ALT (IU/L)	26.8 ± 16.3	24.2 ± 4.5	127.1 ± 173.5	115.5 ± 107.0
TG (mg/dl)	367.2 ± 281.0	92.6 ± 51.7^$^	119.9 ± 52.0	93.1 ± 65.8
T‐Cho (mg/dl)	104.9 ± 30.3	94.9 ± 31.2	250.4 ± 64.1	185.0 ± 71.0**

*Note*: Data represent the mean ± SD. (CE‐2; *n* = 8, HFD; *n* = 22). Abbreviations: ALT, alanine aminotransferase; BW, body weight; GHb, glycosylated hemoglobin; HFD, high‐fat diet; KO, knockout; PG, plasma glucose; T‐Cho, total cholesterol; TG, triglyceride; WT, wild‐type.

* *p* < .05

** *p* < .01 compared with CE‐2 or HFD‐fed WT mice using the Student's *t*‐test.

^$^ *p* < .05.

^$$$^ *p* < .001 compared with CE‐2 or HFD‐fed WT mice using the Aspin–Welch test.

**FIGURE 2:**
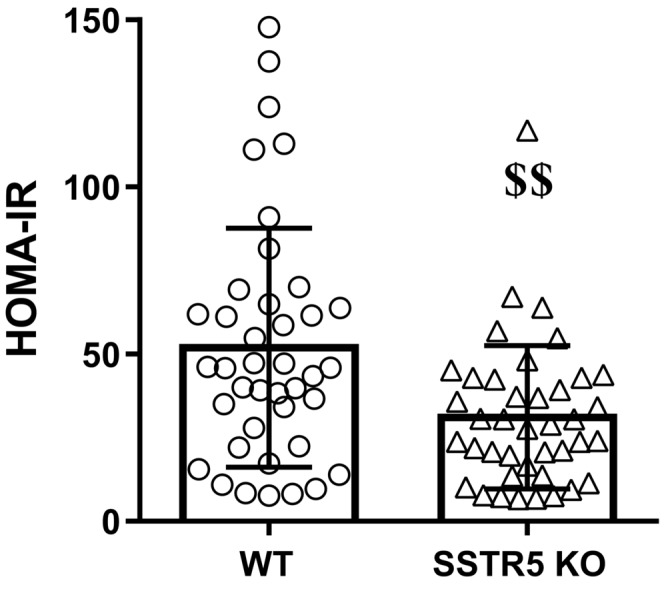
Effects of SSTR5 deletion on homeostasis model assessment of insulin resistance (HOMA‐IR) under high‐fat diet (HFD) feeding condition. Data represent the mean ± S.D. (*n* = 40). ^$$^ *p* < .01 compared with HFD‐fed wild‐type (WT) mice using the Aspin–Welch test. (KO, knockout).

To evaluate the effects of SSTR5 deletion on insulin resistance induced by HFD feeding for 4 weeks, we measured HOMA‐IR, an index of insulin resistance. HOMA‐IR was significantly decreased in HFD‐fed SSTR5 KO mice compared to HFD‐fed WT mice (Figure 2). These results suggest that the deletion of SSTR5 could maintain glucose homeostasis by improving insulin sensitivity.

### 3.2 In vitro profiles of compound‐1, a selective SSTR5 antagonist

The inhibitory activities of compound‐1 against recombinant human and mouse SSTR5 were evaluated. The IC_50_ values of compound‐1 for human and mouse SSTR5 were 9.8 and 31 nmol/L, respectively (Table 2).

**TABLE 2:** Inhibitory activity of compound‐1 against human and mouse SSTR5.

	Human SSTR5		Mouse SSTR5	
	pIC_50_	IC_50_ (nmol/L)	pIC_50_	IC_50_ (nmol/L)
Compound‐1	8.0 (7.8–8.3)	9.8	7.5 (7.3–7.8)	31

*Note*: The pIC_50_ values were determined by nonlinear logistic regression analysis. The 95% confidence intervals of the pIC_50_ values are shown in parentheses. The IC_50_ values were calculated using the following formula: IC_50_ = 10 ^−pIC50^.

To evaluate selectivity to SSTR subtypes, a radioligand binding assay for SSTR1–4 was performed. Compound‐1 showed approximately >1000 times more specificity to SSTR5 than SSTR1–4 (Table 3).

**TABLE 3:** Selectivity of compound‐1 to human SSTR subtypes.

Subtype	Compound‐1
sst1	25%
sst2	<10%
sst3	19%
sst4	66%

*Note*: % Inhibition at a concentration of 10 μmol/L of compound‐1.

### 3.3 SSTR5 antagonist exhibits antidiabetic effects in KK‐A^y^ mice

**FIGURE 3:**
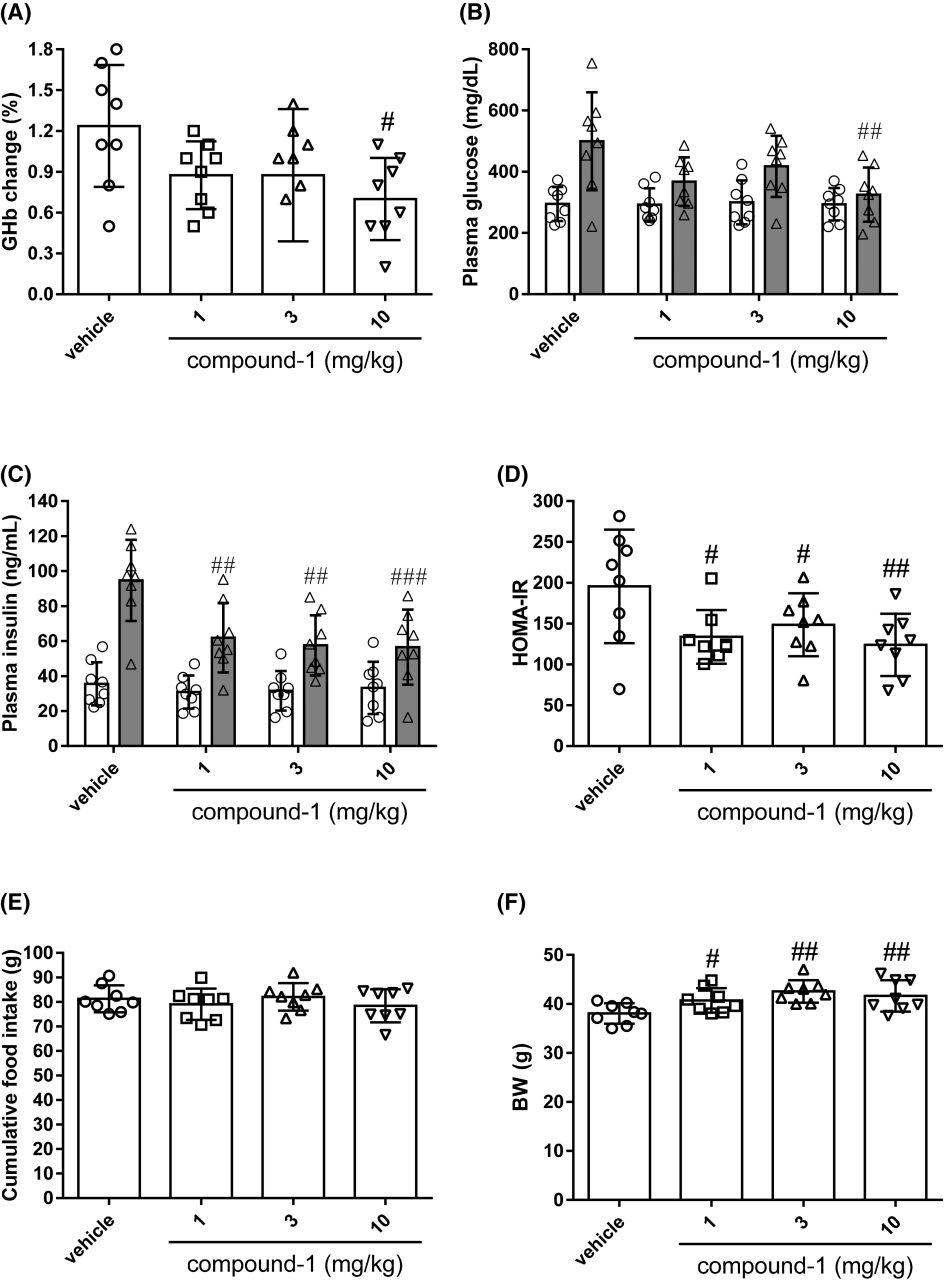
Effects of compound‐1 on diabetic parameters, food intake (FI), and body weight (BW) in KK‐A^y^ mice. Changes in levels of diabetic parameters: (A) glycosylated hemoglobin (GHb), (B) plasma glucose, (C) plasma insulin, and (D) homeostasis model assessment of insulin resistance (HOMA‐IR); (E) cumulative FI, and (F) final BW are shown. Unshaded bar: before drug treatment; shaded bar: after drug treatment (B and C). Data represent the mean ± S.D. (*n* = 8). ^#^ *p* < .05, ^##^ *p* < .01, and ^###^ *p* < .001 compared to the vehicle group using the Williams' test.

To evaluate the therapeutic potential of SSTR5 antagonism for diabetic phenotypes, including insulin resistance, compound‐1 was orally administered to KK‐A^y^ mice at 1, 3, and 10 mg/kg once daily for 2 weeks. In the vehicle‐treated group, the GHb levels increased by 1.24% (from 4.50 ± 0.20% to 5.74 ± 0.53%) after 2 weeks of treatment (Figure 3A); while in the compound‐1‐treated groups (1, 3, and 10 mg/kg), GHb levels increased by 0.88, 0.88, and 0.70%, respectively, after treatment (Figure 3A). In particular, the increase in the 10 mg/kg compound 1‐treated group was 0.54% less than that in the vehicle‐treated group, suggesting the amelioration of the GHb level elevation by compound‐1. Consistent with the reduction in GHb levels, compound‐1 significantly decreased PG, plasma insulin, and HOMA‐IR levels in a dose‐dependent manner (Figure 3B–D). No significant difference was observed in cumulative FI (Figure 3E). BW was slightly increased in the compound‐1‐treated groups (Figure 3F). Measured using an EchoMRI system, the lean mass was significantly increased at ≧1 mg/kg and the fat mass was significantly increased at 10 mg/kg dose (data not shown). These results suggest that the selective SSTR5 antagonist improves insulin sensitivity independent of FI reduction or weight loss in an obese and diabetic animal model.

### 3.4 SSTR5 antagonist improves hepatic insulin sensitivity in KK‐A^y^ mice

**FIGURE 4:**
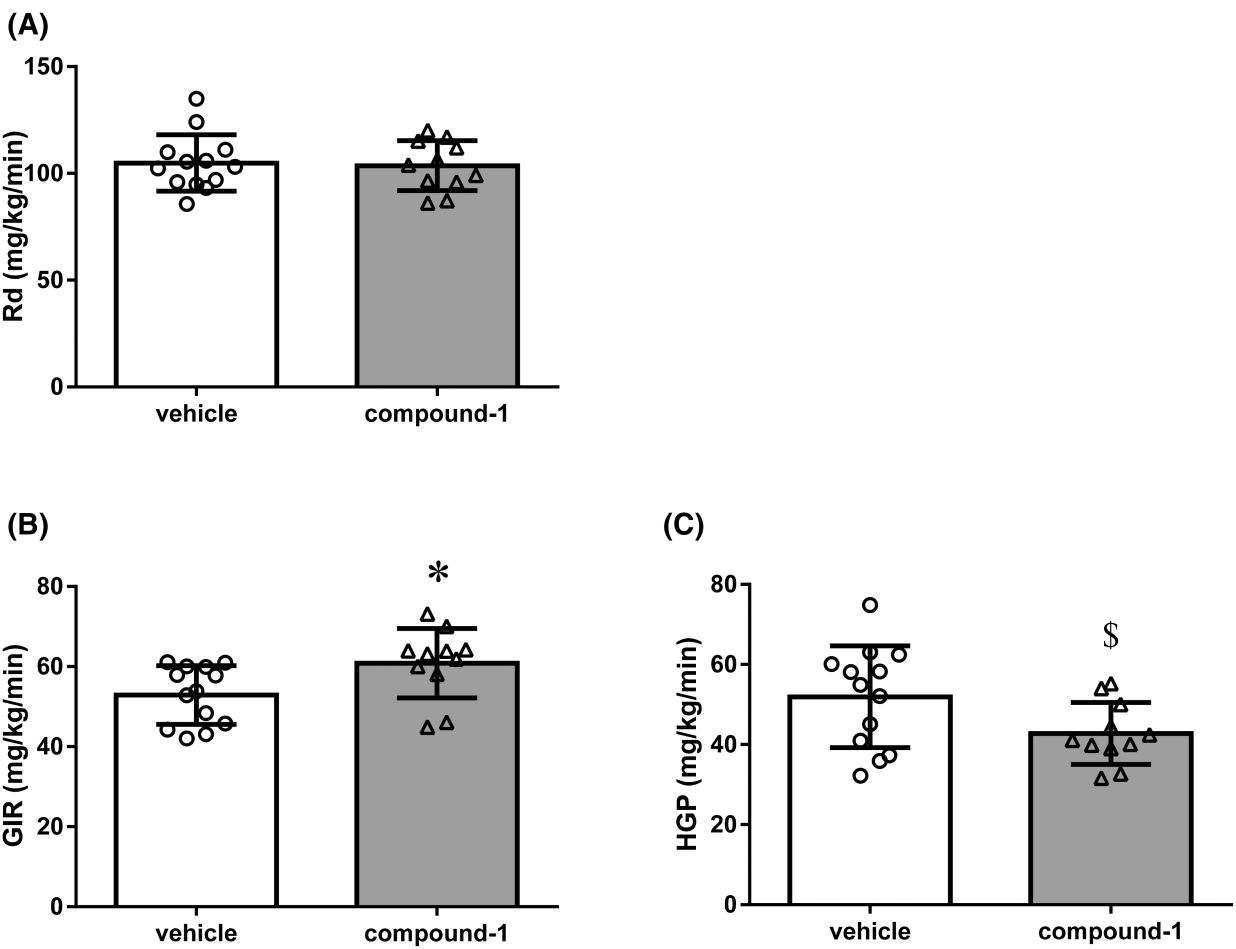
Effects of 0.03% hydrochloride salt of compound‐1 on insulin sensitivity in KK‐A^y^ mice (A) Glucose disappearance rate (Rd), (B) glucose infusion rate (GIR), and (C) hepatic glucose production (HGP) based on hyperinsulinemic‐euglycemic clamp analysis are presented. Data represent the mean ± SD (*n* = 11–13). *p < .05, compared with the vehicle group using the Student's *t*‐test. ^$^ *p* < .05, compared with the vehicle group using the Aspin–Welch test.

The hyperinsulinemic‐euglycemic clamp study was performed in KK‐A^y^ mice under conscious and unrestrained conditions. KK‐A^y^ mice were administered 0.03% hydrochloride salt of compound‐1 for 1 week (39.7 ± 3.0 mg/kg/day equivalent). The change in GHb levels during the 1‐week treatment was 1.15% in the vehicle and 0.49% in the compound‐1 groups, with a difference between the vehicle and compound‐1 groups of 0.66%. The BG level in the compound‐1 group was 0.63 times that in the vehicle group (data not shown). As shown in Figure 4, compound‐1 significantly increased GIR and decreased HGP; however, it did not alter Rd in KK‐A^y^ mice. These results suggest that the selective SSTR5 antagonist improves hepatic insulin sensitivity.

### 3.5 SSTR5 antagonist alleviates the suppression effect of octreotide on insulin‐induced Akt phosphorylation in the liver of C57BL/6J mice

**FIGURE 5:**
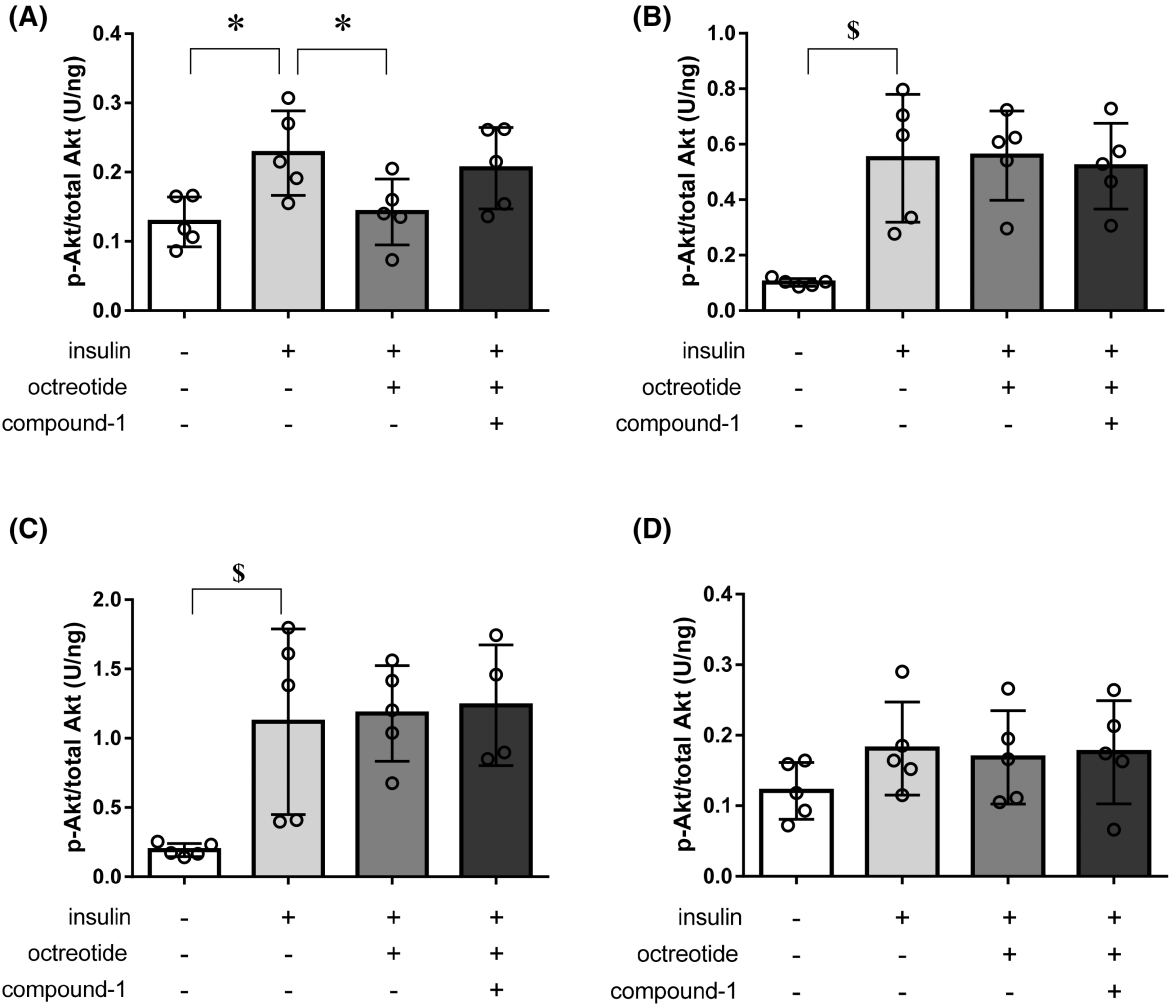
Effect of compound‐1 (10 mg/kg) on insulin‐induced Akt phosphorylation in the tissues of C57BL/6J mice. The ratio of p‐Akt to total Akt in the (A) liver, (B) gastrocnemius muscle, (C) epididymal fat, and (D) subcutaneous fat are presented. Data represent the mean ± SD (*n* = 4–5). *p < .05 using the Student's *t*‐test, ^$^ *p* < .05 using the Aspin–Welch test. A closed procedure was used to keep the overall type‐I error rate at 0.05 (see Statistics section for more details).

To investigate the physiological role of SSTR5 on insulin signals, we evaluated the effect of octreotide, an SST analog that binds to SSTR2 and 5 with high affinity,^41^ and compound‐1 on insulin‐induced Akt phosphorylation in the tissues of C57BL/6J mice. Mice were intraperitoneally administered insulin after octreotide injection with/without oral administration of compound‐1. Insulin administration increased Akt phosphorylation at Ser473 in the liver, gastrocnemius muscle, epididymal fat, and subcutaneous fat by 1.8, 5.4, 5.8, and 1.5 times, respectively, compared to the control group (Figure 5). In the liver, octreotide inhibited insulin‐induced Akt phosphorylation; this effect may have been negated by compound‐1 (Figure 5A). In contrast, neither octreotide nor compound‐1 affected insulin‐induced Akt phosphorylation in other tissues (Figure 5B–D). These results suggest that SST may attenuate insulin signaling in the liver in a tissue‐specific manner, possibly via SSTR5 in mice.

## 4 DISCUSSION

SSTR5 is expressed in the pancreatic β‐cells and intestinal L‐cells; hence, previous studies have suggested that SSTR5 antagonism improves glucose tolerance via insulin and GLP‐1 secretion. Additionally, SSTR5 might regulate insulin sensitivity; however, this has not been elucidated.^35^ Therefore, the current study was performed to determine whether SSTR5 regulates insulin sensitivity in vivo using SSTR5 knockout mice and the selective SSTR5 antagonist.

Obesity is a critical factor for insulin resistance^42^; hence, we investigated whether SSTR5 KO mice are tolerant to obesity‐related insulin resistance induced by an HFD. HFD‐fed SSTR5 KO mice exhibited lower HOMA‐IR than HFD‐fed WT mice at similar BW levels, indicating that SSTR5 deletion maintains insulin sensitivity even under HFD‐induced obesity. In addition, compound‐1 exhibited a glucose‐lowering effect accompanied by an improvement in insulin sensitivity without BW reduction after 2 weeks of treatment in KK‐A^y^ mice. The increase in the lean mass and fat mass in the compound‐1‐treated group could be caused to enhance insulin action in tissues by reduction of hyperglycemia and hyperinsulinemia toxicity by SSTR5 inhibition. The data also indicated that the selective SSTR5 inhibition exhibits antidiabetic effects independent of weight loss, especially by improving insulin sensitivity, in accordance with the phenotype of HFD‐fed SSTR5 KO mice.

Consistent with previous reports, we confirmed that compound‐1 increased plasma GLP‐1 and insulin levels after oral glucose administration in HFD‐fed C57BL/6J mice. To determine whether these hormones participate in the insulin‐sensitizing action of SSTR5 inhibition, we evaluated the effects of alogliptin, a dipeptidyl peptidase 4 (DPP4) inhibitor that increases plasma GLP‐1 and insulin levels, on insulin resistance in KK‐A^y^ mice. Although both compound‐1 and alogliptin suppressed postprandial hyperglycemia in the meal tolerance test in female KK‐A^y^ mice (Figure S1), alogliptin did not reduce HOMA‐IR after 2 weeks of treatment in KK‐A^y^ mice (Figure S2). Consistent with our results, DPP4 inhibitors and GLP‐1 analogs have not been found to significantly improve insulin sensitivity in patients with type 2 diabetes.^43^, ^44^ Therefore, we consider that other mechanisms distinct from the elevation of GLP‐1 and insulin levels contribute to the effects of insulin‐sensitizing action by selective SSTR5 antagonists.

To further investigate the mechanism underlying insulin sensitization by the SSTR5 antagonist, we performed a hyperinsulinemic‐euglycemic clamp under conscious and unrestrained conditions in KK‐A^y^ mice. Although HOMA‐IR is commonly used as a parameter of insulin resistance in terms of convenience, the hyperinsulinemic‐euglycemic clamp can evaluate insulin sensitivity in not only the whole body but also in organs such as the liver, fat, and muscle. Compound‐1 significantly increased GIR and decreased HGP, indicating that an SSTR5 antagonist could increase hepatic insulin sensitivity. In addition, compound‐1 abolished the inhibitory effect of octreotide on insulin‐induced Akt phosphorylation in the liver of C57BL/6J mice. Moreover, the expression of hepatic *G6pc*, involved in HGP, was significantly reduced in HFD‐fed SSTR5 KO mice compared with HFD‐fed WT mice under fasting conditions in our preliminary studies (Figure S3). These data support the findings of the clamp study that the selective SSTR5 antagonist inhibits hepatic insulin sensitivity.

Previous studies have reported that SSTR5 can potentially stimulate phosphotyrosine phosphatases (PTP) activity.^45^, ^46^ In addition, SSTRs, to which octreotide binds, have the potential to dephosphorylate the p85 subunit of phosphatidyl inositol 3 kinase (PI3K) via SHP‐1, a classical PTP activation mechanism, resulting in inhibition of the insulin signal via suppression of PI3K/Akt activity.^47^, ^48^ Considering that both SSTR5 and insulin receptors are expressed in the liver,^22^ an SSTR5 antagonist could rescue the insulin receptor/PI3K/Akt signaling by inhibiting the SSTR5‐stimulated PTP activity in the liver.

Contrastingly, SST and SST analogs decrease the splanchnic blood flow and portal pressure in vivo.^49^, ^50^, ^51^, ^52^ In particular, octreotide and other SST analogs are thought to be useful for the management of gastroesophageal varices and variceal hemorrhage.^53^ The underlying mechanism for SST‐induced vasoconstriction can be elucidated using the phospholipase C/IP_3_ activity/Ca^2+^ mobilization^54^ and PLA2‐dependent arachidonic acid production pathways.^51^ Moreover, the former potency could be the strongest at SSTR5 compared to other SSTR subtypes.^54^ SST28 is secreted after meals,^28^ and SSTR5 is constitutively expressed in the vascular smooth muscle cells,^55^, ^56^ which indicates that SST28/SSTR5 activation causes reduced exposure to insulin and glucose uptake in the liver postprandially. Therefore, the improvement in hepatic insulin sensitivity by the SSTR5 antagonist may be due to the recovery of the SST/SSTR5‐mediated hepatic blood flow decrease through SSTR5 blocking.

A limitation of our study is that we demonstrated that the target organ of SSTR5 antagonists is the liver using a hyperinsulinemic‐euglycemic clamp study and an insulin‐induced Akt phosphorylation model utilizing only one insulin concentration in each of these. However, because each target organ has different sensitivities to the same insulin concentration,^57^ the SSTR5 antagonist causes an increase in insulin sensitivity by the same mechanism in other SSTR5‐expressing tissues, such as skeletal muscles^22^, ^27^, ^58^, ^59^; this assumption could not be denied completely and further investigation is needed.

In conclusion, we demonstrated that SSTR5 antagonists exhibit a glucose‐lowering effect and improve insulin sensitivity in diabetic animal models independent of weight loss. Our results also suggest that the liver is one of the target organs of this mechanism; therefore, selective SSTR5 antagonists may represent a novel and attractive therapeutic agent for type 2 diabetes.

## AUTHOR CONTRIBUTIONS

Research design: Yumiko Okano Tamura, Jun Sugama, Shin‐ichi Abe, Yuji Shimizu, Hideki Hirose, Masanori Watanabe. Experimental procedures: Yumiko Okano Tamura, Jun Sugama, Shin‐ichi Abe, Yuji Shimizu. Data analysis: Yumiko Okano Tamura, Jun Sugama, Shin‐ichi Abe, Yuji Shimizu, Masanori Watanabe. Manuscript writing and editing: Yumiko Okano Tamura, Jun Sugama, Shin‐ichi Abe, Yuji Shimizu, Hideki Hirose, Masanori Watanabe.

## FUNDING INFORMATION

This study was funded by Takeda Pharmaceutical Company Limited.

## CONFLICT OF INTEREST

This work was supported by Takeda Pharmaceutical Company Limited. Among the authors, Yumiko Okano Tamura and Yuji Shimizu are employees of Takeda Pharmaceutical Company Limited. and stockholders of Takeda. Jun Sugama, Shin‐ichi Abe, Hideki Hirose, and Masanori Watanabe were employees of Takeda Pharmaceutical Company Limited. and stockholders of Takeda at the time of their contribution to the study reported.

## ETHICS STATEMENT

The animal experiments were approved by the Institutional Animal Care and Use Committee of Shonan Research Center, Takeda Pharmaceutical Company Limited (Kanagawa, Japan), in a facility accredited by the American Association for Accreditation of Laboratory Animal Care (AAALAC).

## Supporting information


Appendix S1.

## Data Availability

The data that support the findings of this study are available from the corresponding author upon reasonable request.
