# Supplementary material for: Selective somatostatin receptor 5 inhibition improves hepatic insulin sensitivity
Source: Pharmacol Res Perspect. 2022 Dec 30;11(1):e01043. doi: 10.1002/prp2.1043 (PMC9803904; doi:10.1002/prp2.1043)
Supplement: Supplementary file 1 — Appendix S1. [file PRP2-11-e01043-s001.docx]

**Supporting Information**

**Selective somatostatin receptor 5 inhibition improves hepatic insulin sensitivity**

Yumiko Okano Tamura^1^, Jun Sugama^1^, Shin-ichi Abe^1^, Yuji Shimizu^2^, Hideki Hirose^1^, Masanori Watanabe^1*^

^1^Cardiovascular and Metabolic Drug Discovery Unit, Takeda Pharmaceutical Company Limited, Fujisawa, Kanagawa, Japan

^2^Biomolecular Research Laboratories, Takeda Pharmaceutical Company Limited, Fujisawa, Kanagawa, Japan

* Corresponding Author

Masanori Watanabe

SCOHIA PHARMA, Inc., Shonan Health Innovation Park, 26-1, Muraoka-Higashi 2-chome, Fujisawa, Kanagawa, 251-8555, Japan

TEL: +81-70-3875-1079

Fax: +81-466-29-4471

Email: [masanori.watanabe@scohia.com](mailto:masanori.watanabe@scohia.com)

**Supporting Materials and Methods**

***Materials***

Alogliptin benzoate was synthesized by Takeda Pharmaceutical Company Limited (Kanagawa, Japan). Alogliptin benzoate was then suspended in a 0.5% methylcellulose (MC) solution (FUJIFILM Wako Pure Chemical Corporation, Osaka, Japan). The dose of alogliptin was expressed in a free base form.

***Meal tolerance test in KK-A^y^ mice***

Female KK-A^y^ mice aged 13 weeks were used in the study. The animals were divided into three groups (n = 8 in each group) based on glycosylated hemoglobin (GHb), plasma glucose (PG), insulin, and triglyceride (TG) levels, food intake (FI), and change in body weight (BW) during habituation. After overnight fasting, the mice were orally administered vehicle (0.5% MC), compound-1 (1 mg/kg), or alogliptin (30 mg/kg, expressed in a free base form) 1 h before liquid meal challenge (F2LCP, 10.8 kcal/kg, Oriental Yeast Co., Ltd., Tokyo, Japan). BG levels were measured before (t = 0 min) and after meal administration at t = 10, 30, 60, and 120 min via the tail vein using a portable glucose analyzer (Accu-Chek, Roche Diagnostics Corp., Indianapolis, IN, USA).

***Repeated dose study of compound-1 in KK-A^y^ mice***

Female KK-A^y^ mice aged 12 weeks were used in the study. The animals were divided into three groups (n = 8 in each group) based on GHb, PG, insulin, and TG levels, FI, and change in BW during habituation. The mice were orally introduced with vehicle (0.5% MC), compound-1 (1 mg/kg), or alogliptin (30 mg/kg, expressed in a free base form) once daily for two weeks (days 0–13). Finally, on day 14, blood samples were collected from the tail vein of mice under 6 h fasting conditions to measure PG and insulin levels. Homeostatic model assessment for insulin resistance was calculated using the following formula: fasting insulin (μU/mL) × fasting glucose (mg/dL)/405 according to a previously reported method (Matthews 1985).

***Gene expression in high-fat diet (HFD)-fed somatostatin receptor 5 (SSTR5) knockout (KO) study***

For the evaluation of *G6pc* expression under HFD feeding conditions, SSTR5 KO and wild-type (WT) mice were fed an HFD for 16 weeks beginning from 10 weeks of age (n = 7 in each group). At 26 weeks of age, the mice were sacrificed under isoflurane anesthesia under 6 h fasting conditions. Hepatic mRNA levels were measured as follows. Total RNA was isolated from the liver samples that were stored in RNA later using an RNeasy Plus Mini kit (Qiagen, Tokyo, Japan). Reverse transcription reactions were performed using High-Capacity cDNA Reverse Transcription Kit (Thermo Fisher Scientific, Tokyo, Japan), according to the manufacturer’s instructions. Gene expression was quantified using TaqMan real-time PCR (ABI7900; Thermo Fisher Scientific) using TaqMan™ Gene Expression Master Mix and TaqMan® Gene Expression Assays (Thermo Fisher Scientific). The following primer-probe sets were used: glucose-6-phosphatase (*G6pc*, Mm00839363_m1) and β-actin (*Actb*, Mm00607939_s1). The relative gene expression was calculated by the ΔΔCt method and normalized to β-actin expression.

***Statistical analysis***

All data represent the mean ± S.D. Statistical analysis was performed using the SAS systems version 8.2 (SAS Institute Inc., Cary, NC, USA) or EXSUS version 8.0 (CAC Croit Corporation, Tokyo, Japan). To evaluate the effects of drugs in KK-Ay mice, the statistical differences between the vehicle and drug treatments were analyzed by the Dunnett’s test and Steel’s test. To evaluate the effects of SSTR5 deletion *in vivo*, we analyzed the statistical differences between HFD-fed WT mice and HFD-fed SSTR5 KO mice by the Student’s *t*-test.

**Supporting data**

**(B)**

**(A)**


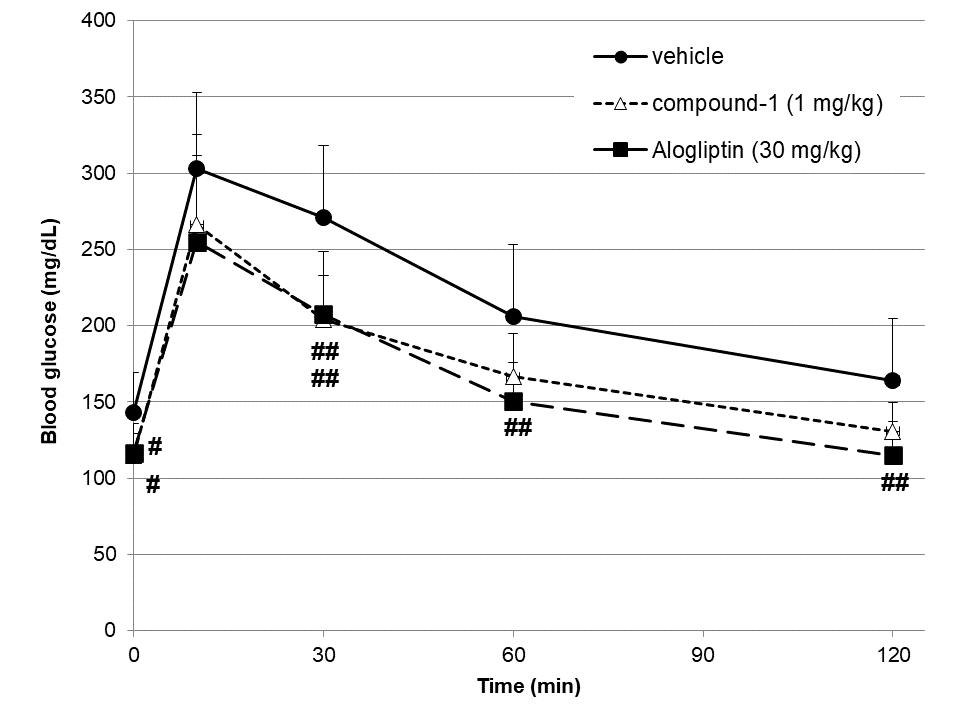


**Figure S1. Effects of a single oral dose of compound-1 or alogliptin on glucose tolerance in a meal tolerance test in KK-A^y^ mice.**

(A) blood glucose (B) and area under the concentration-time curve (AUC)_0-120 min_ of blood glucose levels (B). Data are presented as the mean ± S.D. (n = 8). #*p* < 0.05, ##*p* < 0.01 compared with the vehicle group by the Dunnett’s test.

**Figure S2. Effects of chronic compound-1 or alogliptin on homeostatic model assessment for insulin resistance (HOMA-IR) in KK-A^y^ mice.** Data are presented as mean ± S.D. (n = 8). † *p* < 0.05 compared with the vehicle group by the Steel’s test.

**Figure S3. Effect of SSTR5 on hepatic *G6pc* mRNA expression in high-fat diet (HFD)-fed wild-type (WT) or SSTR5 knockout (KO) mice.** Data are presented as the mean ± S.D. (n = 7). ^#^ *p* < 0.05 vs. WT by the Student’s *t*-test.
